# Central neuraxial anaesthesia presenting with spinal myoclonus in the perioperative period: a case series

**DOI:** 10.4076/1752-1947-3-7293

**Published:** 2009-06-23

**Authors:** Olumuyiwa A Bamgbade, John A Alfa, Wael M Khalaf, Andrew P Zuokumor

**Affiliations:** 1 Department of Anaesthesia, Central Manchester University Hospital, Manchester, M13 9WL, UK; 2 Department of Anaesthesia, Lancashire Teaching Hospital, Preston, PR2 9HT, UK

## Abstract

**Introduction:**

Perioperative spinal myoclonus is extremely rare. Many anaesthetists and perioperative practitioners may not diagnose or manage this complication appropriately when it occurs. This case report of unusual acute spinal myoclonus following regional anaesthesia highlights certain aspects of this rare complication that have not previously been published.

**Case presentations:**

A series of four consecutive patients who developed acute lower-limb myoclonus following spinal or epidural anaesthesia are described. The case series occurred at three different hospitals and involved four anaesthetists over a 3-year period. Two Caucasian men, aged 90-years-old and 67-years-old, manifested unilateral myoclonus. Two Caucasian women, aged 64-years-old and 53-years-old, developed bilateral myoclonus. Myoclonus was self-limiting in one patient, treated with further regional anaesthesia in one patient and treated with intravenous midazolam in two patients. The overall outcome was good in all patients, with no recurrence or sequelae in any of the patients.

**Conclusion:**

This case series emphasizes that spinal myoclonus following regional anaesthesia is rare, has diverse pathophysiology and can have diverse presentations. The treatment of perioperative spinal myoclonus should be directed at the aetiology. Anaesthetists and perioperative practitioners who are unfamiliar with this rare complication should be reassured that it may be treated successfully with midazolam.

## Introduction

Spinal myoclonus is a non-generalized neuromuscular dysfunction that may be focal or segmental, affecting single muscles or muscle groups [1]. It presents as sudden, shock-like bursts of involuntary spasms and usually results from spinal cord pathology such as compression, sepsis, trauma, degeneration, vasculopathy or neoplasm [1]. It may be associated with epilepsy, toxicity, drug reactions, intrathecal analgesics/anaesthetics or intrathecal contrast material [1-3]. This report is a case study and discussion of unusual acute spinal myoclonus following regional anaesthesia. There are very few published reports on this matter, and this clinical report highlights certain aspects that have not been previously discussed in medical literature.

## Case presentations

### Case 1

A 90-years-old Caucasian man presented for prostatectomy. His co-morbidities included atrial fibrillation, cardiac failure, ischaemic heart disease and hypertension, but he had no history of neuropathy. Medications included digoxin, lisinopril, bendrofluazide and nitrates. Laboratory parameters were normal. He made an informed choice of regional anaesthesia. Standard perioperative monitoring included electrocardiography, noninvasive blood pressure and pulse oximetry. Combined spinal/epidural anaesthesia was performed aseptically at the L3/4 space using a 16 G Tuohy needle and a 26 G Whitacre needle. Spinal anaesthesia was established with 2 ml of heavy 0.5% bupivacaine and an epidural catheter inserted for postoperative analgesia. The regional anaesthesia procedure was uneventful. Adequate motor and sensory block was achieved at 10 minutes. Sensory block to the T8 dermatome was confirmed by loss of cold sensation. An initial attempt at lithotomy positioning was associated with right leg myoclonus, which resolved on placing the patient supine. After 10 minutes, repeat lithotomy positioning produced similar myoclonus. Therefore, an epidural dose of 10 ml of 0.25% bupivacaine was administered, with complete resolution of myoclonus after another 10 minutes. Surgery and follow-up after 30 days were uneventful.

### Case 2

A 64-years-old Caucasian woman presented for ureterotomy. Her co-morbidities included bronchitis, coronary artery disease and diabetes mellitus, but she had no history of neuropathy. Medications included salbutamol, nitrates and metformin. The patient made an informed choice of spinal anaesthesia. Standard perioperative monitoring was used. Spinal anaesthesia was performed aseptically at the L3/4 space using a 26 G Whitacre needle and 2 ml of heavy 0.5% bupivacaine. The spinal anaesthesia procedure was uneventful. Adequate motor and sensory block was achieved at five minutes. Sensory block up to the T6 dermatome was confirmed by loss of cold sensation. The intraoperative course was uneventful. After arriving back on the ward 60 minutes after instituting spinal anaesthesia, the patient developed lower-limb myoclonus, lasting less than three minutes and resolving spontaneously. There was no recurrence of myoclonus and no sequelae. Subsequent spinal block with bupivacaine three months later was uneventful.

### Case 3

A 53-years-old Caucasian woman presented for pelvic surgery. Her co-morbidities included hypertension and rheumatoid arthritis but no neuropathy. Medications included oestradiol, bendrofluazide and dexamethasone. Previous regional block and general anaesthesia were uneventful. Serum vitamin B12 level was 191 ng/L (normal range 200-900 ng/L), but other laboratory parameters were normal. The patient made an informed choice of spinal anaesthesia. Standard perioperative monitoring was used. Spinal block was performed aseptically at the L3/4 spinal space with a 25 G Whitacre needle and 2.5 ml of heavy 0.5% bupivacaine plus diamorphine 300 mcg. The spinal block procedure was uneventful. Adequate motor and sensory block was achieved at 5 minutes. Sensory block up to the T6 dermatome was confirmed by loss of cold sensation. The intraoperative course was uneventful. However, after returning to the ward, about 90 minutes after instituting spinal block, she developed lower-limb myoclonus. The myoclonus lasted one hour and was treated with intravenous midazolam 4 mg. Subsequent follow-up for two weeks revealed no sequelae and no recurrence of myoclonus.

### Case 4

A 67-years-old Caucasian man presented for refashioning of a left below-knee amputation stump. His co-morbidities included diabetes, cardiovascular disease, peripheral neuropathy, obesity and asthma. Medications included insulin, amlodipine, lisinopril, gabapentin and salbutamol. Previous regional block and general anaesthesia were uneventful. The patient made an informed choice of spinal anaesthesia. Standard perioperative monitoring was used. Spinal block was performed aseptically at the L4/5 spinal space with a 25 G Whitacre needle and 2.5 ml of heavy 0.5% bupivacaine plus diamorphine 300 mcg. The spinal block procedure was uneventful. Adequate motor and sensory block was achieved at five minutes. Adequate sensory block up to the T8 dermatome was confirmed by loss of cold sensation. The intraoperative course was uneventful. However, at the end of surgery, about 60 minutes after the spinal blockade, the patient complained of repetitive muscle spasm in the absent amputated limb. The phantom spasm was distressful to the patient and was promptly treated with intravenous midazolam 5 mg. There was no recurrence of myoclonus and no sequelae.

## Discussion

Spinal myoclonus is rare and usually involves muscles innervated by adjacent spinal-cord segments [1]. The pathophysiology of spinal myoclonus includes abnormal loss of inhibition from suprasegmental descending pathways, loss of inhibition from local dorsal horn interneurons, hyperactivity of contiguous anterior horn neurons, and aberrant local axon re-excitations [1,2]. Loss of local or suprasegmental inhibitory function in the spinal cord may account for the myoclonus in this report. Myoclonus following regional anaesthesia is extremely rare, and there are only very few reports [3,4]. This case series occurred at three different hospitals and involved four anaesthetists over a three-year period. Rapid onset of myoclonus less than 90 minutes after spinal anaesthesia, as in our report, is extremely unusual. Previous reports recorded later onset times of many hours or days [3,4]. There is no obvious explanation for the extremely acute onset of myoclonus in our patients. The pathophysiology may be related to the neurotoxic effect of local anaesthetics or opioids and local neuronal irritation by spinal needle or catheter.

Spinal myoclonus may result from high-dose spinal, epidural or systemic opioid therapy [2,5,6]. High doses of spinal opioid combined with spinal-cord or nerve degeneration are risk factors [5]. Intravertebral disease is associated with increased risk of myoclonus because of the combination of spinal-cord/nerve dysfunction and high intrathecal or systemic opioid analgesia requirement. The pathophysiology of myoclonus following high-dose opioid analgesia may involve an imbalance between the activity of spinal and central opioid receptors or direct opioid toxicity on the medulla spinalis [5].

The management of opioid-induced spinal myoclonus involves change of opioid, dose reduction or change from intrathecal to systemic administration. Intrathecal dose reduction may be combined with systemic administration to improve the imbalance between spinal and central opioid receptor activity [5]. None of the patients in this report received high doses of intrathecal opioid, and the two patients who did receive intrathecal opioid were administered a low dose of diamorphine 300 mcg. Therefore, these cases of spinal myoclonus are unlikely to be opioid-induced. However, it is possible that low doses of intrathecal opioid may contribute to spinal myoclonus in the presence of spinal-cord or neuromuscular dysfunction.

Local anaesthetic neurotoxicity may cause spinal myoclonus, and there have been some debate and reports regarding this adverse effect [3,4,7]. However, bupivacaine was the local anaesthetic administered to the patients in this report, and it has a good safety record especially at low doses of less than 13 mg, such as administered to these patients [7]. Although unlikely, it is possible that the occurrence of myoclonus was associated with local anaesthetic neurotoxicity in the presence of subtle underlying neuropathy.

Spinal myoclonus may result from local neuronal irritation or injury by the intrathecal needle or catheter. Spinal-cord or nerve injury presents with pain during the regional anaesthesia procedure and subsequent acute neuropathy, with or without myoclonus, which may be unilateral or bilateral [1,3,8]. Indwelling spinal or epidural catheters may cause myoclonus by irritating the spinal cord or nerve roots, and this complication can be treated by withdrawing the offending catheter [9]. Procedural neurologic injury may precipitate spinal myoclonus by causing abnormal impulse transmission or aberrant hyper-excitability in the spinal nerve root, with consequent disturbance of spinal inhibitory neurons and hyper-excitability of anterior horn cells. Sympathetic hyper-excitability may also contribute to spinal myoclonus [1,8]. There was no obvious neurologic trauma during the regional block procedure for our patients, because the procedure was uneventful and comfortable for all the patients. However, subtle neurologic irritation may not manifest significant pain, but may aggravate pre-existing neuropathy and result in perioperative myoclonus such as in Case 4. Three patients denied pre-existing neuropathy, but it is possible that they had some degree of undiagnosed subtle pre-existing neuropathy, especially Patient 1 who was 90-years-old with risk of neuro-degeneration and Patient 2 who had diabetes with risk of neuropathy.

Unilateral spinal myoclonus following regional anaesthesia, such as in Case 1 and Case 4, is extremely rare. A previous report involved epidural needle injury followed by rapid-onset spinal myoclonus, which was relieved by lumbar plexus block [8]. The patient in Case 1 developed unilateral myoclonus following spinal block, which was relieved by further regional anaesthesia in the form of epidural block. Although it may seem somewhat ironic, the administration of further regional anaesthesia is an effective means of treating myoclonus following regional block, possibly by correcting the disinhibition and hyperactivity in the spinal cord [6,8]. Another report of unilateral myoclonus involved intrathecal catheter irritation of spinal nerve root, with occurrence of spinal myoclonus whenever the patient stood up from a seated position; this was treated by catheter withdrawal [9]. The myoclonus in Patient 1 occurred at initial attempts to place the patient in lithotomy position. This may be associated with neuronal irritation by the indwelling epidural catheter and/or stretching of the spinal nerves during hip flexion. There is a report of amputation-stump myoclonus [10], but our report of phantom-limb spasm in Patient 4 is unique and not previously reported. We believe this phantom spasm was due to loss of inhibition from descending spinal-cord pathways.

Vitamin deficiency syndromes may predispose to neuropathy and perianaesthesia myoclonus [11,12]. A possible contributory factor to the myoclonus in Patient 3 is the mild vitamin B12 deficiency. Vitamin B12 deficiency is associated with myelopathy and neuropathy [11]. However, the degree of deficiency in our patient was mild and she did not require vitamin B12 therapy for the resolution of myoclonus. Lower-limb neuropathy after spinal block has been reported in a case of thiamine deficiency [12].

Chronic use of certain medications may predispose to spinal myoclonus. The patients in Case 1 and Case 3 were on chronic diuretic therapy, with the risk of electrolyte disorder which may predispose to neurologic dysfunction. However, none of the patients had electrolyte derangements. The patient in Case 3 was on chronic dexamethasone and oestradiol therapy, and chronic steroid therapy is associated with neuropathy [13]. Although difficult to prove, this may be a contributory factor to the spinal myoclonus in Patient 3.

The treatment of spinal myoclonus involves detection of the aetiology, abolition or minimisation of the aetiology, and symptomatic treatment with benzodiazepines, baclofen, anticonvulsants or serotoninergics [1,14]. If treatment of the underlying disorder is impossible, symptomatic treatment is worthwhile, although limited by side-effects and lack of controlled evidence. Intrathecal baclofen is effective therapy for spinal myoclonus [1] but was not attractive to our team and patients because of the route of administration and adverse central neurologic effects. Anticonvulsants such as carbamazepine, topiramate and sodium valproate are also effective [10,14].

Benzodiazepines are effective anticonvulsants and should constitute the mainstay of treatment for myoclonus [14]. Diazepam and clonazepam have been used successfully. Midazolam was used for treatment in two of our patients because it is readily available in ready-to-use, injectable form. It is highly potent, painless on injection, rapid-onset, with predictable effect and duration [14]. Thus, we believe that midazolam is the benzodiazepine of choice for treating perioperative spinal myoclonus. It is also arguable that the low incidence of perioperative myoclonus may be related to the relative use of midazolam for premedication.

## Conclusion

Acute spinal myoclonus following regional anaesthesia is extremely rare, and treatment should be directed at the aetiology. Anaesthetists should watch out for this anaesthetic complication, especially in patients with underlying vitamin deficiency or neuromuscular disease. Anaesthetists who are unfamiliar with this rare complication should be reassured that it may be treated successfully with midazolam. Extra caution should be taken when administering intrathecal opioid or anaesthetic in patients with known or suspected neuromuscular dysfunction. Long-term follow-up is vital to monitor for recurrence or sequelae.

## Consent

Written informed consent was obtained from the patients for publication of this case report and any accompanying images. A copy of the written consents is available for review by the Editor-in-Chief of this journal.

## Competing interests

The authors declare that they have no competing interests.

## Authors' contributions

All the authors made substantial contributions to the conception of this report, were involved in writing the manuscript, read it and approved it to be published.
